# Human papillomavirus genotype distribution in cervical cancer biopsies from Nepalese women

**DOI:** 10.1186/s13027-018-0176-7

**Published:** 2018-01-17

**Authors:** Sunil Kumar Sah, Joaquin V. González, Sadina Shrestha, Anurag Adhikari, Krishna Das Manandhar, Shyam Babu Yadav, David A. Stein, Birendra Prasad Gupta, María Alejandra Picconi

**Affiliations:** 1 B. P. Koirala Memorial Cancer Hospital, Bharatpur, Chitwan Nepal; 2 Oncogenic Viruses Laboratory, National Institute of Infectious Diseases-ANLIS “Dr. Malbrán”, Av. Velez Sarsfield 563, C1282AFF Buenos Aires, Argentina; 3 National and Regional HPV Reference Laboratory, National Institute of Infectious Diseases-ANLIS “Dr. Malbrán”, Av. Velez Sarsfield 563, C1282AFF Buenos Aires, Argentina; 4 Kathmandu Research Institute for Biological Sciences, Lalitpur, Nepal; 5 Department of Health Service, Ministry of Health, Government of Nepal, Kathmandu, Nepal; 6 Central Department of Biotechnology, Tribhuvan University, Kirtipur, Kathmandu, Nepal; 7 Department of Biomedical Sciences, Oregon State University, Corvallis, Oregon, USA

**Keywords:** Human papillomavirus, Cervical cancer, HPV genotyping, Nepal

## Abstract

**Background:**

Cervical cancer (CC) is the leading cause of morbidity and mortality from cancer in Nepalese women. Nearly all cases of CC are caused by infection with certain genotypes of human papillomavirus (HPV). Data on HPV genotype distribution in Nepalese CC patients is sparse. We aimed to determine the distribution of HPV genotypes in biopsies of CC tissue from Nepalese women.

**Methods:**

This study examined 248 archived paraffin-embedded tissue specimens from CC cases from patients of B.P. Koirala Memorial Cancer Hospital, Bharatpur, Chitwan, Nepal. DNA was extracted from the biopsies and HPV detection performed by PCR. HPV genotyping was then carried out by a reverse line hybridization technique capable of identifying 36 distinct HPV genotypes.

**Results:**

Most of the samples were from tumors that had been designated by hospital pathologists as squamous cell carcinoma (77.6%). 165 of the 248 samples contained DNA of sufficient quality for rigorous PCR testing. All the analyzable specimens were positive for HPV. The most common HPV genotypes, in decreasing order of frequency were 16, 18, 45, 33, 52, 56 and 31; most were found as single infections (94.5%). Together, HPV types 16, 18, and 45 were found in 92% of the tumor samples.

**Conclusion:**

This study strengthens the knowledge-base of HPV genotype distribution in CC cases in Nepal. Hopefully, this information will be useful to the medical community and public health policy-makers in generating improved HPV-surveillance, −prevention and -treatment strategies in Nepal.

## Background

In 1983, Harald zur Hausen and colleagues isolated human papillomavirus (HPV)-16 and 18 from human cervical cancer (CC) tissues and helped define the central role of HPV in the development of CC [1]. HPV is now recognized as the etiologic agent causing almost all invasive CC and a major carcinogen causing other human malignancies as well, including vulvovaginal, oropharyngeal, penile, and anal cancers [2].

Currently, within the family *Papillomaviridae*, more than 200 HPV genotypes have been characterized [3]. Over 40 genotypes, all in the *Alphapapillomavirus* genus, can infect the female and male anogenital region. Infection with some of the genotypes can cause benign genital warts while others cause precursor cervical lesions, cervical intraepithelial neoplasia (CIN) and CC [4]. Of the 40, at least 12 HPV genotypes have been definitively associated with progression of CIN to CC and are considered carcinogenic to humans [5]. HPV genotypes 16, 18, 31, 33, 35, 39, 45, 51, 52, 56, 58, and 59 are classified as carcinogenic to humans (Group 1; often referred to as high-risk, HR), HPV-68 as probably carcinogenic to humans (Group 2A), HPV-types 26, 30, 34, 53, 66, 67, 69, 70, 73, 82, 85 and 97 (Group 2B) as possibly carcinogenic to humans, while HPV-6 and 11 (Group 3; often referred to as low-risk, LR) are not currently classifiable as to their carcinogenicity to humans [6]. Of the HR-HPV-genotypes, genotypes 31, 33, 35, 52 are phylogenetically related to HPV-16 while genotypes 39, 45, 59, and probably 68 are more closely related to HPV-18 [7].

Worldwide, CC is the fourth most common type of cancer among women, and causes over 40% mortality [8]. Currently, more than 80% of new CC cases are diagnosed in lower- and middle-income countries such as Nepal, India, Bangladesh and Sri Lanka [9]. Annually, in Nepal, over 2000 new cases of CC are diagnosed and over 1000 deaths are attributed to CC [10]. However, due to the lack of both a national screening program for CC and a reliable national database for cancer cases in general, the actual number of cases and deaths probably exceeds those reported estimates [11].

Nepal is located in Southern Asia, adjacent to both India and China, in the Himalayan region. Along with indigenous peoples, many of Nepal’s inhabitants are descendants of migrants from India, southern China, Myanmar, Tibet and other areas of Central Asia, making Nepal a multiethnic and multicultural country. Nepal’s population has been steadily rising in recent decades, currently reaching approximately 26 million (census 2011). As in most developing countries, a spectrum of sanitary and public health conditions are present, due at least in part to transportation limitations and reduced access to medical services in rural compared to urban areas.

Several studies have addressed the prevalence and genotypes of HPV circulating in Nepalese females having non-cancerous cytology, as well as in those having preneoplastic and neoplastic cervical lesions [9]. However, current and comprehensive data regarding HPV genotype distribution in Nepalese CC cases is scarce.

Although HPV-16 and 18 are the predominant HPV genotypes associated with CC worldwide, at least 25% of CC cases are associated with other HPV genotypes [12]. Differences in the relative prevalence of the various CC-associated HPV genotypes has been documented to occur both regionally and across more large-scale geographic areas [13].

Three prophylactic HPV vaccines are currently available worldwide: a quadrivalent vaccine was first licensed in 2006, a bivalent vaccine in 2007 and a nonavalent vaccine in 2014. All protect against infection with HPV-16 and 18; while the most recent also protects against five additional HR-HPV types (HPV-31, 33, 45, 52, and 58). Current evidence suggests the three vaccines offer comparable efficacy in prevention of CC. The choice of HPV-vaccine is typically based on data regarding a number of factors, including the type and scale of local HPV-associated pathologies, the target-populations for which the vaccine has been approved and product characteristics (e.g. cost, availability) [14].

The goal of this study was to determine the distribution of HPV-genotypes found in CC cases of Nepalese women. We are hopeful that this information will be useful to public-health officials for designing improved strategies for the prevention of HPV-infection and its related diseases.

## Methods

### Study design

A retrospective cross-sectional study was designed and coordinated by the BP Koirala Memorial Cancer Hospital (BPKMCH), Bharatpur, Chitwan, Nepal, in collaboration with the Central Department of Biotechnology, Tribhuvan University, Kritipur, Nepal, Kathmandu Research Institute for Biological Sciences, Lalitpur, Nepal, and the National Institute of Infectious Diseases (INEI) -ANLIS “Dr. Malbrán”, Buenos Aires, Argentina. The study was approved by the Nepal Health Research Council.

Archived formalin-fixed paraffin-embedded sections of tumor tissue biopsies that had been excised from CC patients (30–99 year old women) during 2011 to 2014 at BPKMCH were examined. A random sampling method was used for tissue specimen selection and de-identification was done prior to specimen handling, to avoid any sampling-bias. Information regarding the age at collection, year of collection, and histological findings generated from the samples was obtained.

### Fixed and paraffin embedded cervical tissue block processing

All cervical tumor tissue blocks were processed at INEI under stringent laboratory conditions to avoid contamination of or between samples. For each ten case-block of samples, a paraffin blank-block was also sectioned and included, as a control for contamination. One to five 10 μm sections of tissue from each sample were transferred to a 1.5 mL screw-cap tubes using a fresh tooth pick for each section, for use in downstream techniques described below. Fresh gloves were used for each case. After the sectioning of each case the scalpel used was cleaned with R-WAX (BioPack, Argentina). A blank-block was sectioned after each ten case-block.

### DNA extraction

DNA was extracted from the sectioned blocks using a method developed at the United States Centers for Disease Control (US CDC) using the QIAamp DNA Blood MiniKit (QIAGEN) [15]. Briefly, 180 μl of ATL buffer was added to a microcentrifuge tube containing a single tissue section and heated at 120 °C for 20 min, which melted the paraffin. At approximately 5 min into the heating step, the tube contents were mixed by gentle tapping. After heating, the samples were incubated at room temperature for 3 min, followed by quick centrifugation. Twenty microliters of proteinase K was added to the tube, followed by brief vortexing, then incubated at 65 °C for 16 h [15]. The tubes where then briefly centrifuged and 200 μl ATL buffer and 200 μl ethanol was added, yielding a final volume of around 400 μl. After a brief vortex the mixture was loaded into a QIAamp Mini spin column and centrifuged at 8000 rpm for 1 min. The elution steps were performed according to the manufacturer’s protocol except the elution volume was 50 μl AE buffer pre-warmed to 55 °C.

### HPV detection and genotyping

HPV detection was performed using PCR with biotinylated Broad-Spectrum General Primers (BSGP) 5+/6+ designed to amplify a highly conserved 140 bp fragment of the HPV-L1 gene [16]*.* Genotyping was carried out by a reverse hybridization line which identifies 36 HPV- genotypes (6,11,16,18,26,31,33,34,35,39,40,42,43,44,45,51,52,53,54,55,56,57,58,59,61,66,68,70,71,72,73,81,82,83,84,89). Briefly, the denaturated biotynilated amplicons, obtained from amplification of sample DNA with BSGP5+/GP6+ primers, were hybridized with genotype-specific oligonucleotide probes immobilized as parallel lines on membrane strips (Reverse Line Blot Hybridization, RLB). The hybrids were treated with alkaline phosphatase-streptavidin conjugate and substrate (ECL Detection Reagents) resulting in a chemiluminescent product subsequently detected by exposure to autoradiography film. The β-globin gene was co-amplified and its relative abundance detected in each sample, to serve as an internal control [17]. The INEI laboratory, which performed the HPV detection and genotyping in this work, is the HPV Regional Reference Laboratory for the Americas (within the Global HPV Laboratory Network (Global HPVLabNet)) and annually participates in an international Global HPV DNA typing proficiency study [18].

### Data analysis

Data analysis was performed using Epi Info version 3.5.3 (US CDC). In determining the frequency of HPV genotypes, each sample was scored for the genotype detected and, if more than a single genotype was detected, for the combination of genotypes detected.

## Results

**Fig. 1:**
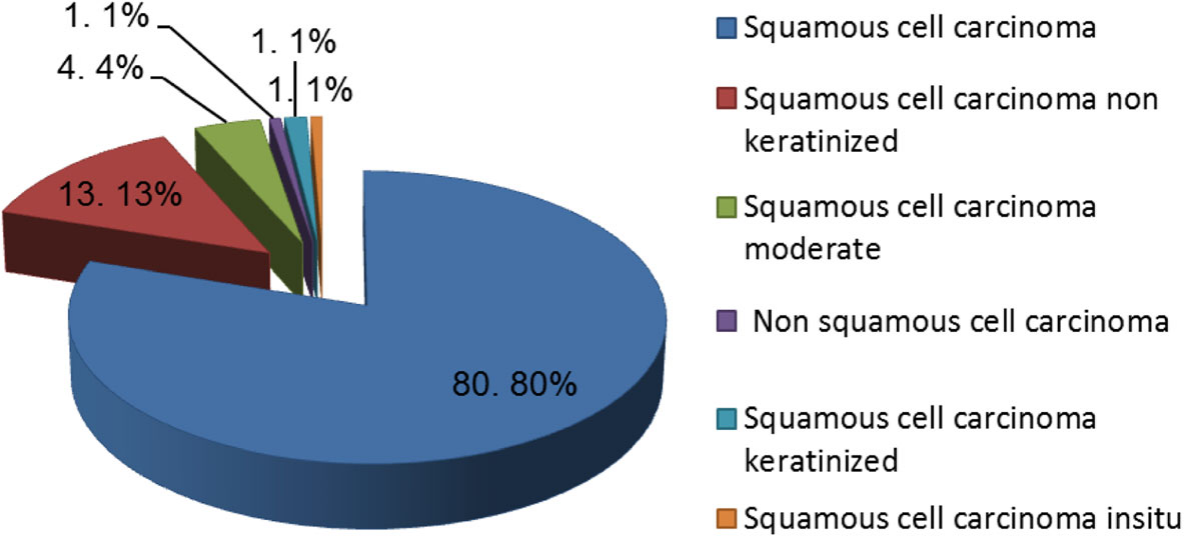
Histological diagnosis of cervical cancer analyzed in the study

Of the 248 paraffin embedded CC tissue blocks originally selected, the β-globin gene was amplifiable in only 165, thus our further analysis was restricted to this subset. Histological analysis of these samples revealed that 81 of the 165 blocks contained squamous cell carcinoma (Fig. 1).

All the 165 analyzed blocks were positive for HPV; among them, 156 (94.5%) were infected with a single genotype and 9 (5.5%) were infected with multiple genotypes. Almost all cases were positive for at least one HR-HPV genotype, and one sample harbored HPV-6 (Table 1). The HPV genotypes identified belong to one of five species of the fifteen total species of the α-papillomavirus genus: α5 (HPV-51; 0.5%), α6 (HPV-56; 2.3%), α7 (HPV-18, 39, 45 and 68; total: 20.7%), α9 (HPV-16, 31, 33, 35 and 52; total: 75.9%), and α10 (HPV-6; 0.5%).

**Table 1:** Distribution of HPV genotypes in cervical cancer from Nepalese women

HPV Genotype	Samples (*n* = 165)	Single infection (a)	Multiple infection (b)
HR-HPV	Frequency (%)	Frequency (%)	Frequency (%)
16	119 (72, 2)	111 (93.3)	8 (6.7)
18	25 (14,8)	22 (88.0)	3 (12.0)
31	3 (1.8)	2 (66.7)	1 (33.3)
33	4 (2,4)	4 (100)	0 (0)
35	2 (1,2)	2 (100)	0 (0)
39	2 (1,2)	1 (50)	1 (50)
45	8 (4,8)	6 (75)	2 (25)
51	1 (< 1)	0 (0)	1 (100)
52	4 (2,4)	2 (50)	2 (50)
56	4 (2,4)	4 (100)	0 (0)
68	1 (< 1)	1 (100)	0 (0)
LR-HPV			
6	1 (< 1)	1 (100)	0 (0)

The most common genotypes detected, in decreasing order of frequency, were HPV-16, 18, 45, 33, 52, 56, and 31. HPV-16, 18, and 45 were the three most prevalent types in all histological groups (squamous cell carcinoma, adenocarcinoma, and adenosquamous cell carcinoma). One or more of these three genotypes were detected in 152/165 (92%) of the tumor tissue samples.

Samples testing positive for HPV-18 or 45 were from women of a higher age than those infected with HPV-16 (Table 2). The mean ages of women with invasive CC associated with HPV-16, 18 and 45 were 45–57 years (95% CI 43.5–47.5), 55.4 years (50.3–60.6) and 52.3 years (44.1–60.5), respectively, while the average age of those with CC associated with any other HPV genotype was higher, at 56 years [53.9–58.1].

**Table 2:** Distribution of HPV genotypes (single and multiple infections) in invasive cervical cancer according to age among Nepalese women

HPV genotype	Age (Years) Frequency (%)				Total Frequency (%)
	< 40	40–49	50–59	> 60	
6	0 (0%)	0 (0%)	1 (100.0%)	0 (0%)	1 (100%)
16	7 (6.3%)	27 (24.3%)	42 (37.9%)	35 (31.5%)	111 (100%)
18	1 (4.5%)	7 (31.8%)	6 (27.3%)	8 (36.4%)	22 (100%)
31	0 (0%)	1 (50.0%)	0 (0%)	1 (50.0%)	2 (100%)
33	0 (0%)	1 (25.0%)	0 (0%)	3 (75.0%)	4 (100%)
35	0 (0%)	1 (50.0%)	1 (50.0%)	0 (0%)	2 (100%)
39	0 (0%)	0 (0%)	0 (0%)	1 (100.0%)	1 (100%)
45	0 (0%)	2 (33.3%)	2 (33.3%)	2 (33.3%)	6 (100%)
52	1 (50.0%)	0 (0%)	0 (0%)	1 (50.0%)	2 (100%)
56	0 (0%)	0 (0%)	2 (50.0%)	2 (50.0%)	4 (100%)
68	0 (0%)	0 (0%)	1 (100.0%)	0 (0%)	1 (100%)
16 + 18	1 (33.3%)	0 (0%)	1 (33.3%)	1 (33.3%)	3 (100%)
16 + 39	0 (0%)	0 (0%)	0 (0%)	1 (100.0%)	1 (100%)
16 + 45	0 (0%)	0 (0%)	2 (100.0%)	0 (0%)	2 (100%)
16 + 51	0 (0%)	0 (0%)	1 (100.0%)	0 (0%)	1 (100%)
16 + 52	0 (0%)	1 (0%)	0 (0%)	0 (0%)	1 (100%)
31 + 52	0 (0%)	1 (0%)	0 (0%)	0 (0%)	1 (100%)

## Discussion

This study defines the HPV-genotype distribution in the largest series of CC tissue samples from Nepal addressed to date. We analyzed archived biopsy specimens from the B.P. Koirala Memorial Cancer Hospital, one of the few hospitals in Nepal specializing in cancer diagnosis and treatment. Patients from throughout Nepal are referred to this hospital, thus the samples analyzed can be considered as representative of the Nepalese population overall.

In our study, HPV detection was performed by PCR using the BSGP5+/6 + multiplexed with-globin system, which is technically superior to the original GP5+/6+ PCR system [16], and suitable for large-scale epidemiological studies. The PCR system we used is designed to amplify fragmented DNA and thus the detection method of choice with archived formalin-fixed, paraffin-embedded tissues samples, which are prone to degradation from fixative-induced cross-linking [19].

In agreement with previously published studies on CC in local, regional and worldwide populations, we found that squamous cell carcinoma was the dominant CC histo-type (77.6%) [12].

Our results are consistent with previous studies documenting HPV-16 as the most common and HPV-18 as the second-most common genotypes associated with CC worldwide. We found HPV-16 and 18 to be associated with 87% of the total CC tissue samples in our study, consistent with a previous study of 54 Nepalese cases detecting HPV-16/18 in 90% of samples [20] . Our study detected HPV-16/18 at a moderately higher rate than previously reported for Southern Asia (80%) [21], and the Asian region (71%) in a worldwide study [12]**,** but a little smaller than those rates from Eastern India that exceeds 100%, considering multiple infections (HPV-16: 83.78%; HPV-18, 21.08%) [22].

HPV45 was the next most common genotype found in our study (4.8%), in agreement with previous publications on local and regional populations [12, 20]. Together the three most-common HPV genotypes (HPV-16, 18 and 45) were found in over 90% of our samples. Our results vary somewhat from a previous study which detected HPV-58 in 4.0% of Asia-wide samples [12] whereas here, with Nepal-only samples, HPV-58 was not detected at all. As well, HPV-56 was present in < 1% of samples in the Asia-wide data while in our study it was detected in 2.4% of samples.

The obtained results confirmed the inverse correlation between HPV genotype diversity and progressive disease [23]. Also in Nepal, the genotype distribution in normal cytology reveals a wide spectrum of HPV types, both low and high risk types, without marked predominance of none of them (HPV-70, 4.7%; HPV-16, 1.4%; HPV-58, 0.9%; HPV-56, 0.7%; HPV-18, 0.6%; HPV-52 y 39, 0.4%; HPV-35, 0.3%; HPV-33 y 45, 0.2%) [24–26]; as the severity of the cervical lesion increases, HR-HPV genotype become the most frequent types, being almost the only ones in CC, with a remarkable majority of HPV-16 and HPV-18, as it was shown in our study.

Most of the samples examined in this study were infected with only one HPV genotype, which is consistent with the previously observed inverse correlation between HPV diversity and neoplastic lesion [19]. Moreover, our findings are consistent with the ecological principles of competitive exclusion and carcinogenesis hallmarked by clonal expansion and evolution of transformed cells [27, 28].

Our detection of HPV-6 in one CC biopsy containing no other HPV-genotypes is a rare and noteworthy event. According to the IARC-WHO Working Group Reports, the carcinogenic potential of HPV-6 and 11 is considered “not classifiable”, a category which includes agents that are considered to have low carcinogenic potential, based on the available epidemiological and experimental data [28]. The rationale for this categorization of HPV-6 and 11, rather than the “probably not carcinogenic” classification, is the low but established probability of finding either genotype associated with a small percentage (0.45% [95% CI: 0.35–0.56]) of CC cases worldwide. It has been postulated that HPV-6 and other low-risk genotypes may in rare cases cause cancer as a result of unusual “virus-host circumstances” [29].

The results from this and previous studies documenting the presence of HPV-16 and 18 in a high percentage of CC tissue samples represent further evidence that implementation of an HPV vaccine program designed to address at least these two HPV genotypes could significantly lower the incidence of CC in Nepal. Fortunately, all three HPV vaccines provide considerable cross-protection against numerous disease-associated HPV types not specifically included as antigenic targets in the respective vaccine formulations. Based on evidence from clinical trials and post-introduction impact evaluations, the bivalent and quadrivalent HPV vaccines provide protection against HR-HPV genotypes other than HPV-16 and 18, such as HPV-31, 33 and 45, all of which have been implicated to cause preneoplastic lesions and subsequent cancer [30].

Numerous previous studies have made similar optimistic predictions regarding the potential of HPV-vaccination to greatly reduce the overall number of cervical abnormalities [14, 31]. Preceding studies in developing countries have documented that HPV vaccination supplemented with regular screening is a highly cost-effective strategy to reduce the incidence of and mortality from cervical cancer [32].

## Conclusion

This study increases our knowledge of HPV genotype distribution in cervical cancer cases from Nepal. In anticipation that Nepal will soon implement more effective widespread public health measures to prevent HPV infections and their sequelas, particularly CC, it is important to have epidemiologic data regarding the prevalence of HPV genotypes. Considering the high prevalence of HPV-16 and 18 (83%) found in the CC from Nepalese women in this study, we expect that the widespread introduction of any of the approved HPV-vaccines will sharply decrease the incidence of CC in Nepal. Considering the cross-protection generated against most or all of the disease-associated HR-HPV genotypes by the currently-marketed vaccines, including the relatively inexpensive bivalent vaccine [30, 33], widespread vaccination with any of the current HPV vaccines is expected to have a multiplicity of benefits to Nepalese public health. It is our hope that this study will be useful to Nepalese public-health officials in generating improved strategies for prevention of HPV infection and its associated diseases, as well as pre- and post-vaccine surveillance.

## Abbreviations

BPKMCH: B. P. Koirala memorial cancer hospital
BSGP: Broad-Spectrum General Primers
Ca: cancer
CC: cervical cancer
CDC: center for disease control
CI: confidence interval
CIN: cervical intraepithelial neoplasia
GHPVLN: Global HPV laboratory network
HPV: human papillomavirus
HR: high risk
ICC: invasive cervical cancer
LR: low risk
SCC: squamous cell carcinoma
